# Congenital pouch colon in Duhok, outcome and complications: Case series

**DOI:** 10.1016/j.amsu.2019.07.031

**Published:** 2019-08-01

**Authors:** Qadir Mohammed Salih Qadir, Ayad Ahmad Mohammed

**Affiliations:** a Pediatric Surgeon, University of Duhok, College of Medicine, Duhok Pediatric Surgery Center, Duhok City, Kurdistan Region, Iraq; b General Surgeon, University of Duhok, College of Medicine, Department of Surgery, Azadi Teaching Hospital, 8 Nakhoshkhana Road, 1014 AM, Duhok City, Kurdistan Region, Iraq

**Keywords:** Congenital colon pouch, Anorectal malformations, Window colostomy, Colostomy, Ileostomy

## Abstract

**Background:**

Congenital pouch colon (CPC) is a rare congenital abnormality associated with anorectal malformations with high incidence of complications and mortality. The aim of this study is to describe the various types of congenital colon pouch, their management aspects, complications of surgery, and the best management options.

**Results:**

The incidence of congenital pouch colon in the present study was 5.3% (18 patients) of all anorectal malformations. Sixteen cases (88.8%) were males and 2 cases (12.5%) were females, (M: F ratio was 8:1). The age of presentation was ranged from 1day to 1year; 17 cases were presented in first week of life. Preoperative diagnosis of congenital pouch colon was done in 7 patients. As an initial procedure tabularization of the pouch with end colostomy was done in 15 cases, window colostomy was done in 2 cases, and excision of the pouch and proximal ileostomy was done in one patient. As a definitive procedure, abdomino-perineal pull-through of the tabularized pouch was done in 15 cases, ileo-anal anastomosis after pouch excision was done in 3 cases.

**Conclusions:**

Pouch tabularization and end colostomy had better outcome than other types of interventions. Abdomino-perineal pull through of the tabularized pouch was the definitive surgical procedure for treatment of complete pouch colon in our study.

## Highlights

• Congenital pouch colon is a rare disease that requires early diagnosis and treatment.
• Window colostomy at the pouch is better to be avoided due to high incidence of complications.
• Pouch tabularization and end colostomy has better outcome than other types of interventions.

## 1 Introduction

Congenital pouch colon (CPC) is a very rare type of anorectal malformation, in this condition there is huge segmental dilatation of variable length of the colon which is communicated distally by a fistulous tract with the genitourinary system [[1], [2], [3], [4]].

The incidence of this condition is estimated to be 10–15% of patients with congenital anorectal anomalies and this condition is commoner in males than females [4].

The condition is associated with other anomalies of various systems of the body such as the genitourinary system in the form of hydro-ureter, hydro-nephrosis, hypoplasia of the kidney, uterine anomalies, cloaca, colo-vestibular fistula, colo-vesical fistulae, or bladder extrophy [[4], [5], [6], [7], [8]].

Most affected patients present in the early neonatal period with absent anus and intestinal obstruction. Female patients may present with passage of the meconium from abnormal opening in the perineum or cloaca, such group of patients usually present later [4].

Most cases can be diagnosed with an erect skiagram and the conventional invertogram done for patients with anorectal anomalies.Plain abdominal X-ray may be valuable to determine the type of the of the pouch alleviating the need for the invertogram [4,9].

The work of this case series has been reported in line with the PROCESS 2018 criteria [10].

## 2 Methods

This article had been registered in accordance with the declaration of Helsinki at the Research registry.com in the 24th of April 2019, the registration number is 4829, ethical approval was gained also from the Duhok Directorate General of Health at the 12th of March 2019 with reference number 12032019-2.

This is a case series which is a retrospective study included cases of complete congenital pouch colon. The study is a single center experience conducted in Duhok pediatric surgery center in Heevi Pediatrics Teaching Hospital, College of Medicine, University of Duhok, Duhok city, Kurdistan region, north of Iraq.

Data collected in the period between the 14th of August 2014 to the 1st of March 2017. During this period 18 consecutive cases with congenital pouch colon who presented to the hospital were included in this study, the majority of them were among the refugees of the camps.

The participants were from the pediatric age group presented with CPC malformation, many of them had other associated congenital anomalies such as congenital heart diseases, urogenital malformations and other various congenital malformations.

At admission the initial management consisted of placing the patient in the incubator to treat hypothermia, correction of fluid and electrolyte disturbances, decompression of the stomach and bowels through nasogastric tube, administration of 0.1 mg/kg vitamin K as a single dose intravenously, and intravenous antibiotics.

Abdominal and the perineal examinations performed by a pediatric surgeon to detect the site of fistula and abnormalities of the sacrum and the perineal muscles. General examination was performed to exclude other associated congenital malformations. Plain skiagram of abdomen and invertogram were the initial and sole investigations which were done for 15 cases. Ultrasonography of abdomen and pelvis were also done in 15 cases and echocardiography in 12 cases.

The surgical procedures were divided into primary surgical procedure which was done as an emergency surgery and definitive surgical procedure which was done to reconstruct the bowel continuity and manage any associated fistula. During the primary surgical procedure careful intraoperative evaluation was done to define the anatomical abnormalities. The definitive surgical procedure was done between the fifth to the sixteenth months of age.

All the operations were performed by a specialist pediatric surgeon with a surgical experience in dealing with congenital colorectal malformations for the last 10 years.

Regular follow up was done for 14 cases for one year to assess the postoperative outcome and to diagnose any postoperative complication. The weight of the patient and fecal continence were evaluated at each follow up visit.

## 3 Results

The incidence of congenital pouch colon in the present study was 5.3% (18 patients) of all anorectal malformations. A total number of 18 cases were included in this case series. Sixteen cases (88.8%) were males and 2 cases (12.5%) were females, (M: F ratio was 8:1). The age of presentation was ranged from 1day to 1year; 17 cases were presented in first week of life. Ten cases had absent normal colon and the terminal ileum was opened into the abnormal colonic pouch and the majority of them presented with a good general condition because they presented early less than 24 h from delivery due to acute presentation except one female who presented at the age of one year. Table 1.

**Table 1:** Preoperative categorization of patients with congenital pouch colon.

Patient categories:							
Group	Weight; kg	General condition	Presentation	Sepsis	Complications	Patients number	Percentage
**I**	>2.5	Good	<24hr	Absent	Absent	10	55.5
**II**	>=2.5	Fair	<=24hr	Present	_ Absent	6	33.3
**III**	<2.5	Poor	>24hr	Present	Present	2	11.1

All the male cases had an absent anal opening and were presented with abdominal distention, 12 of them had history of passing of meconium in urine, whereas the female cases (2 cases) one of them was presented with the passage of meconium from a vestibular fistula and the other one from a single perineal opening (cloaca). Two cases were preterm.

The prenatal history in 11 cases showed that the mother has history of genitourinary tract infections and 10 of them had history of drug ingestion, the family history was positive for the same condition in only one case. Table 2.

**Table 2:** Prenatal history in patients with congenital pouch colon.

Prenatal History:		
	Frequency	Percentage
Prenatal conditions		
Infections (UTI, PID)	11	61.1
Drug ingestion	10	55.5
Polyhydramnios	7	38.8
Exposure to radiation X-ray	3	16.6

**Fig. 1:**
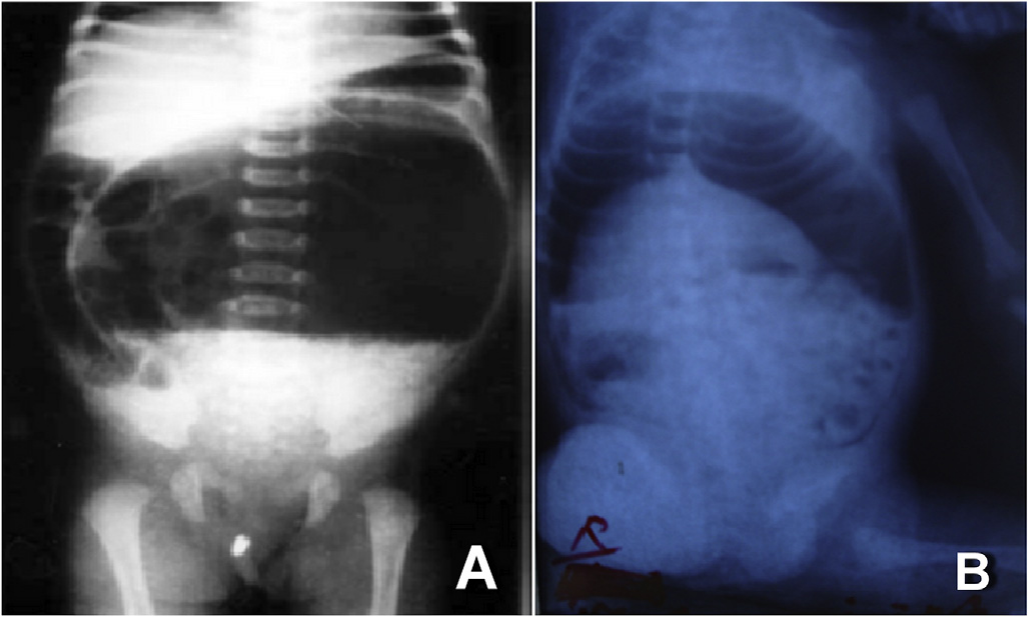
Plain abdominal X-ray taken preoperatively showing a large air fluid level(A) and free air under both doms of the diaphragms (B).

Abdominal X-ray showed a large air fluid level in 5 cases (27.7%), multiple air fluid levels in 4 cases (22.2%), free air in the peritoneal cavity in 2 cases (11.1%), double bubble appearance in one case and was not significant in the remaining. Fig. 1.

In 15 cases there was a fistula which was connected to the urinary system and in the remaining 3 cases the pouch was ending blindly or attached to the urinary bladder by fibrous band. The external anal sphincter and muscle complex were well formed in most cases, while in those with high type anorectal malformation the anal sphincter was poorly developed. Table 3.

**Table 3:** Associated anomalies that discovered preoperatively and intraoperatively.

Associated congenital anomalies:		
Associated Malformation		
Bilateral vesicoureteric reflux	4	26.6
Cyanotic congenital heart disease	4	26.6
Sacral agenesis	4	26.6
Left hydronephrosis	3	20
Left kidney agenesis	2	13.3
Right undescended testis	2	13.3
Absent appendix	2	13.3
Mega cystitis	1	6.6
Hypospadias (distal shaft)	1	6.6
Malposition of right kidney	1	6.6
Duodenal atresia	1	6.6
Fistula Presence Intraoperatively		
Urinary bladder	9	50
No fistula (Blind end or fibrous)	3	16.6
Vestibule	1	5.5
Prostatic urethra	1	5.5
Common channel cloaca	1	5.5
External anal sphincter		
Normal	12	66.6
Weak	6	33.3
Muscle complex		
Normal	11	61.1
Weak	7	38.8
Sacrum		
Normal	14	77.7
Atrophied	4	22.2

The primary surgical procedure consisted of pouch tabularization and end colostomy which was done for 15 (83.3%) cases, while the most common definitive surgical procedure was abdomino-perineal pull through of the tabularized pouch. Table 3.

During surgery tabularization of the pouch with end colostomy after ligation of fistula with the urinary bladder was performed in 15 cases, window colostomy was done in 2 cases who had bowel perforation, and ileostomy was done for one case after excision of pouch. Table 4.

**Table 4:** Type of the surgical procedures done for the patients.

Type of the surgical procedure:		
Surgical procedures	Frequency	Percentage
Primary surgical procedure		
Pouch tabularizing and end colostomy	15	83.3
Window colostomy	2	11.1
Pouch excision, Ileostomy	1	5.5
Definitive surgical procedure		
Abdomino-perineal pull through of tabularized pouch	15	83.3
Abdomino- perineal pull through of the ileum	3	16.6

**Fig. 2:**
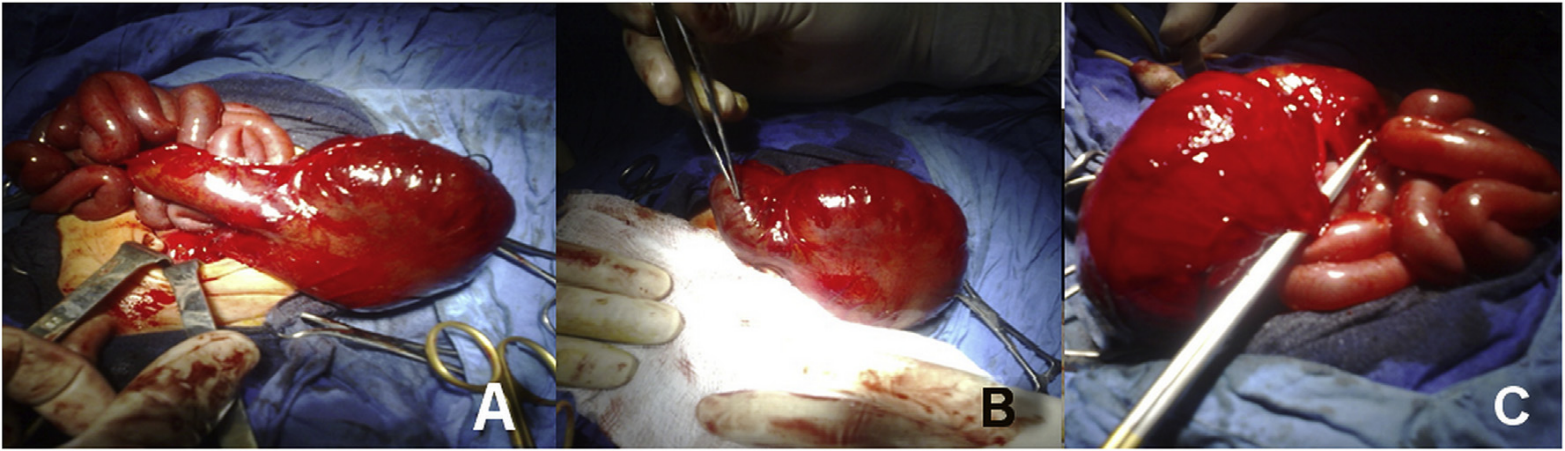
Intraoperative pictures showing, The insertion of fistula was very close to the entrance of ileum to the pouch (A) and (C) and abrupt transition from proximal normal bowel to the pouch(C).

In cases of wide fistula, the pouch had thick wall and tabularization was easier than cases with absent or narrow fistula where the pouch had a very thin wall and was easily perforated. The inferior mesenteric artery was absent in all the cases and the pouch was supplied by a single branch from the superior mesenteric artery. Fig. 2 and Table 3.

Early postoperative complications included stoma related complications in 12 cases, urinary tract infection in 3 cases and wound infection in 2 cases. Late complications included failure to thrive, anal stenosis, fecal incontinence, and other complications. Table 5.

**Table 5:** Complications during follow up at one year.

Clinical follow up:		
Follow up	Frequency	Percentage
Clinical follow up Failure to thrive	5	27.7
Anal stenosis	4	23.1
Anal mucosal prolapse	4	23.1
Fecal incontinence	3	16.6
Re dilatation	3	16.6
Constipation	1	5.5
**Death**	3	16.6
U/S finding during follow up a		
Normal (no abnormal finding)	4	26.6
Bilateral hydroureter	4	26.6
Left hydronephrosis	3	20
Left kidney agenesis	2	13.3
Malrotated right kidney	1	6.6
Lowe abdominal mass (hydrocolpus)	1	6.6

a Ultrasound follow-up done for 15 patients.

Histopathology reported the presence of mature ganglion cells, hypertrophied nerve bundles and giant cells (more than 6 nuclei). In all cases the muscle coats were not differentiated to inner circular and outer longitudinal but instead they were arranged in a decussating pattern, the mucosa and submucosa were normal.

During clinical follow up three cases died, from pneumonia due to associated duodenal atresia, cyanotic congenital heart disease, and ileostomy retraction and intra-abdominal sepsis.

## 4 Discussion

This condition was first described in 1912 by Spriggs in London Hospital Museum. Later and in 1959, Trusler described the condition clinically. Many criteria should be present to diagnose CPC such as the presence of anorectal agenesis, short total colonic length, colonic pouch of variable length, abnormal blood supply of the pouch, thick wall and muscular colonic wall, fistula between the colon and the genitourinary tract, and the absence of transitional zone between the normal and abnormal colonic segments [4].

The pathogenesis of this rare anomaly is not yet well understood but theories suggest that distal obstruction from any cause may be the most important cause, other authors suggest an early vascular insult from obliterated inferior mesenteric artery which leads to failure of the hindgut development which may be the cause of this condition. Many other factors have been found to be associated with the development of this condition such as environmental factors, genetic and dietary factors like iodine and vitamin B12 deficiency. About 90% of the cases are reported from India but recently there is increasing awareness about this rare anomaly and some cases are now reported from other parts of the world [4,[11], [12], [13]].

The most accepted and widely used classification system of CPC is that based on the length of the normal colon that is present proximal to the abnormal segment. In this classification 4 types can be described, type I: normal colon is not present and the terminal ileum opens into the abnormal colon pouch, type II: the terminal ileum opens to the cecum which in turn opens to the colonic pouch, type III: there is a significant normal colonic segment between the ileum and the colonic pouch, and type IV: the colon is almost normal and the pouch involves the sigmoid and the rectum. Some authors describe a 5th type which is extremely rare and only few cases had been reported which consists of 2 colonic pouches with an intervening normal colonic segment [14,15].

The aim of surgery is to preserve the ileocecal valve when possible, preserve as much possible of colon by tabularizing the pouch, and to provide a continent anal opening. The treatment options are excision in the incomplete types and coloplasty in complete types of CPC. Colostomy when done is usually part of the primary procedure. The surgery can be done laparoscopically successfully [13,16,17].

The most important step in the management is proper resuscitation, bowel decompression, correction of fluid and electrolytes abnormalities, prevention of hypothermia, antibiotics, and vitamin K administration [4].

We did pouch tabularization and end colostomy in the majority of our cases (15 cases), while window colostomy was done for 2 cases only. Complete excision of the pouch with end colostomy is the procedure of choice when possible, but many surgeons do window colostomy which is very simple and may be appropriate in emergency and critical presentations [4].

In most of the cases the histopathological examination shows non-organized muscle layers with normal ganglion cell in the bowel wall, the presence of abnormal ganglion cells will mandates complete excision of the pouch, but many surgeons do not perform biopsy during the primary surgical procedure such as colostomy [8,11].

Early post-operative care is aimed at preventing stenosis of the new formed anus by the mean of regular dilatation, making the stool soft and preventing perineal skin excoriation [8].

In most of our patients there were colostomy related complications such as skin excoriation in 12 of them, colostomy prolapse in 2 cases, ileostomy retraction and intra-abdominal sepsis and the patient died from this event. Most authors document that stoma related complications with urinary tract infections are the most common complications [4,18].

Other complications may include stenosis of the anus, mucosal prolapse, dilatation of the retained pouch with fecal overloading, psychological and sexual problems [8].

Three of our patients died from different causes, the mortality and the overall prognosis depends on the weight and the general condition of the patients at presentation, and the presence or absence of other congenital anomalies. Sacral agenesis, bilateral vesicoureteric reflux and cyanotic congenital heart diseases were the most common associated congenital anomalies. The mortality rate may reach 10–15% after surgical intervention [19,20].

All authors agree in that there is no primary single procedure for CPC and the staged procedure is tailored for individual patients, patients may have variable associated congenital malformation. Patients presenting with peritonitis, longer and more complex malformations, and patients with cardiac malformations have poor outcomes [21].

The long term outcomes of the CPC appear to be dismal regardless the type of the surgical intervention that has been done, even if the sacrum is normal and the anorectal muscles are well developed [22].

## 5 Conclusions

The present article is focusing on the high incidence of complications and the possible mortality with ileostomy and colostomy in treating patients with congenital pouch colon at the first operation. Pouch tabularization and end colostomy has better outcome than other types of interventions as a primary procedure. Abdomino-perineal pull through of the tabularized pouch is the best definitive surgical procedure for treatment of complete pouch colon.

Future studies should focus on the possible prenatal diagnosis and larger number of patients should be included from different geographical areas, prenatal diagnosis when possible allow earlier intervention and possible better outcomes.

## Conflicts of interest

No conflict of interest present.

## Source of funding

There is no source of funding other than the authors.

## Provenance and peer review

Not commissioned externally peer reviewed.

## Ethical approval

Ethical approval was gained from the Duhok Directorate General of Health at the 12th of March 2019 with a reference number 12032019-2.

## Author contribution

Study design: Dr Ayad Ahmad Mohammed.

Data collections: Dr Qadir Mohammed Salih Qadir.

Data analysis: Dr Qadir Mohammed Salih Qadir and Dr Ayad Ahmad Mohammed.

Writing: Dr Qadir Mohammed Salih Qadir and Dr Ayad Ahmad Mohammed.

Final approval of the manuscript: Dr Qadir Mohammed Salih Qadir and Dr Ayad Ahmad Mohammed.

## Research registration number

4829 Research registry, at 24/4/2019.

## Trial registry number

N/A.

## Guarantor

Dr Ayad Ahmad Mohammed.
