# Supplementary material for: Congenital pouch colon in Duhok, outcome and complications: Case series
Source: Ann Med Surg (Lond). 2019 Aug 1;45:86–90. doi: 10.1016/j.amsu.2019.07.031 (PMC6690573; doi:10.1016/j.amsu.2019.07.031)
Supplement: Multimedia component 1 [file mmc1.docx]

**Patient categories:**

| Group | Weight; Kg | General condition | Presentation | Sepsis | Complications | Patients number | Percentage |
| --- | --- | --- | --- | --- | --- | --- | --- |
| I | >2.5 | Good | <24hr | Absent | Absent | 10 | 55.5 |
| II | >=2.5 | Fair | <=24hr | Present | _ Absent | 6 | 33.3 |
| III | <2.5 | Poor | >24hr | Present | Present | 2 | 11.1 |

Table 1: Preoperative categorization of patients with congenital pouch colon.

***Prenatal History:***

|  | Frequency | Percentage |
| --- | --- | --- |
| Prenatal conditions  Infections (UTI, PID)  Drug ingestion  Polyhydramnios  Exposure to radiation X-ray | 11  10  7  3 | 61.1  55.5  38.8  16.6 |

Table 2: Prenatal history in patients with congenital pouch colon.

**Associated congenital anomalies:**

| Associated Malformation  Bilateral vesicoureteric reflux  Cyanotic congenital heart disease  Sacral agenesis  Left hydronephrosis  Left kidney agenesis  Right undescended testis  Absent appendix  Mega cystitis  Hypospadias (distal shaft)  Malposition of right kidney  Duodenal atresia | 4  4  4  3  2  2  2  1  1  1  1 | 26.6  26.6  26.6  20  13.3  13.3  13.3  6.6  6.6  6.6  6.6 |
| --- | --- | --- |
| Fistula Presence Intraoperatively  Urinary bladder  No fistula (Blind end or fibrous)  Vestibule  Prostatic urethra  Common channel cloaca | 9  3  1  1  1 | 50  16.6  5.5  5.5  5.5 |
| External anal sphincter  Normal  Weak  Muscle complex  Normal  Weak  Sacrum  Normal  Atrophied | 12  6  11  7  14  4 | 66.6  33.3  61.1  38.8  77.7  22.2 |

Table 3: Associated anomalies that discovered preoperatively and intraoperatively.

***Type of the surgical procedure:***

| Surgical procedures | Frequency | Percentage |
| --- | --- | --- |
| Primary surgical procedure  Pouch tabularizing and end colostomy  Window colostomy  Pouch excision, Ileostomy | 15  2  1 | 83.3  11.1  5.5 |
| Definitive surgical procedure  Abdomino-perineal pull through of tabularized pouch  Abdomino- perineal pull through of the ileum | 15  3 | 83.3  16.6 |

Table 4: Type of the surgical procedures done for the patients.

***Clinical follow up:***

| Follow up | Frequency | Percentage |
| --- | --- | --- |
| Clinical follow up  Failure to thrive  Anal stenosis  Anal mucosal prolapse  Fecal incontinence  Re dilatation  Constipation  Death | 5  4  4  3  3  1  3 | 27.7  23.1  23.1  16.6  16.6  5.5  16.6 |
| U/S finding during follow up *  Normal (no abnormal finding)  Bilateral hydroureter  Left hydronephrosis  Left kidney agenesis  Malrotated right kidney  Lowe abdominal mass (hydrocolpus) | 4  4  3  2  1  1 | 26.6  26.6  20  13.3  6.6  6.6 |
| *Ultrasound follow-up done for 15 patients. | | |

Table 5: Complications during follow up at one year.
